# Does Teacher Support Scaffold Engagement? Academic Self-Efficacy as Mediator and Proactive Personality as Moderator Among Chinese High School Students

**DOI:** 10.3390/bs15111594

**Published:** 2025-11-20

**Authors:** Wenmei Sun, Qiaoyu Wu, Xinle Zhang, Daixin He, Xubo Liu

**Affiliations:** Faculty of Education, Henan Normal University, Xinxiang 453007, China; 2410283138@stu.htu.edu.cn (Q.W.); 1910284097@stu.htu.edu.cn (X.Z.); 2410283124@stu.htu.edu.cn (D.H.); 19100274046@stu.htu.edu.cn (X.L.)

**Keywords:** perceived teacher support, academic self-efficacy, proactive personality, academic engagement

## Abstract

The current study investigates how high school students’ perceived teacher support relates to their academic engagement, drawing on Job Demands–Resources (JD-R) and Conservation of Resources (COR) Theories. It further examined academic self-efficacy’s mediating role and the moderating role of proactive personality. The study used Perceived Teacher Support Behavioral Questionnaire, Academic Self-Efficacy Scale, Proactive Personality Scale, and Academic Engagement Scale to survey 1664 students at a public high school in a county-level city in southeastern Henan Province, China. Findings showed that (1) perceived teacher support positively influenced students’ academic engagement; (2) academic self-efficacy partially mediated this connection; (3) proactive personality moderated the indirect effect, specifically the path from academic self-efficacy to academic engagement. In conclusion, academic self-efficacy acts as a key factor through which perceived teacher support promotes students’ academic engagement. Proactive personality further strengthens the effect of academic self-efficacy on engagement.

## 1. Introduction

Academic engagement contributes to both students’ academic performance and mental well-being (Zhou et al., 2022; Wong et al., 2024). Specifically, it is associated with students’ future academic achievement and increases their life satisfaction (Lewis et al., 2011; Rooij et al., 2017). Academic engagement refers to an individual’s sustained mental state in the learning process, characterized by persistence when encountering difficulties and the attainment of positive self-experience (Schaufeli et al., 2002b). The effort required to maintain this engagement can be seen as a demand. Individuals will experience fatigue when confronting demanding tasks unless sufficient corresponding resources are provided to offset these demands as mentioned in Job Demands–Resources (JD-R) Model (Bakker et al., 2005). In the academic context, secondary school students face more intensified academic assignments and challenging knowledge that often causes increased stress (Z. H. Liu, 2016). Excessive pressure diminishes students’ positive self-related experiences and reduces their willingness to maintain academic engagement. However, perceived teacher support, as a critical protective resource, mitigates the negative impacts of stressors (e.g., academic pressure) on students (García-Moya et al., 2023). Therefore, it is hypothesized that perceived teacher support may boost students’ academic engagement.

Clarifying the core mechanisms underlying perceived teacher support’s contribution to academic engagement of students provides educators with practical strategies so that they can better guide students to enhance their engagement throughout high school.

### 1.1. Perceived Teacher Support and Academic Engagement

The teaching process is inherently reciprocal, involving both teachers and students. Therefore, its effectiveness hinges on both teachers’ behaviors and students’ awareness of that. In this sense, perceived teacher support is conceptualized as the support teachers offer concerning their academic learning, abilities, and emotions (Babad, 1990; OuYang, 2005). When students perceive strong support, a sense of competence and relatedness emerges, which further fosters greater academic engagement (Jin & Wang, 2019; Strati et al., 2017).

In Ecological Systems Theory (Bronfenbrenner & Evans, 2000), schools function as micro-systems that influence students’ development. In school, teachers serve as primary facilitators of education. Their behavioral guidance and emotional care act as protective supportive factors in students’ learning. Such supports alleviate students’ academic pressure and burnout, thereby enhancing their well-being (Fall & Roberts, 2012; Sun et al., 2024) and motivate them to invest more psychological resources in learning.

This perspective is complemented by Shumaker and Brownell’s (1984) Social Support Theory, which emphasizes that access to social supportive resources improves an individual’s ability of environmental adaptation. In the classroom, teachers represent a primary source of such support. Teachers’ supportive behaviors have been shown to boost students’ academic resilience (Reddy et al., 2003; Ahmed et al., 2010). By fostering it, teacher supports help them better bounce back from academic challenges, adapt seamlessly to classroom learning dynamics and facilitate their engagement. Empirical evidence from a diary study by Robayo-Tamayo et al. (2020) confirms that day-to-day perceptions of teacher support can effectively promote individuals’ academic engagement.

Therefore, the first hypothesis posits that:

**H1:**  *There is a positive correlation between high school students’ perceived teacher support and their academic engagement.*

### 1.2. Academic Self-Efficacy’s Mediating Role

Students’ academic self-efficacy, as defined by Bandura (1986), denotes learners’ firm conviction in their capacity to successfully accomplish their academic objectives and to achieve expected academic outcomes, which is regarded as a key source of learning motivation.

Given Expectancy-Value Theory (Wigfield & Eccles, 2000), individuals’ perceived competence in completing tasks is a pivotal factor in determining the extent to which they engage in and persist with tasks. Within educational settings, such perceived competency manifests specifically as academic self-efficacy. It enables students to comprehend their academic potential and pushes them to devote efforts to their studies. In other words, students with better self-efficacy will invest more effort in their learning activities, strengthening heightened academic engagement.

Empirical studies under various research backgrounds have shown consistent positive correlation between academic self-efficacy and academic engagement. Learners who possess high academic self-efficacy show higher interest in English classroom tasks (Cong et al., 2024). In addition, students show more zeal in their math learning if they hold a positive belief in their ability (Olivier et al., 2019).

As posited in Self-Determination Theory by Deci and Ryan (2016), effective social environmental support satisfies individuals’ basic psychological needs, thereby enhancing motivation. When teaching, teachers’ supportive behaviors make students feel qualified and autonomous, which intrigues their intrinsic motivation. Specifically, when students receive encouragement or assistance from teachers, their need for competence and autonomy is fulfilled, stimulating their inner motivation and thus boosting their confidence in completing academic tasks—academic self-efficacy.

Empirical studies have shown that adolescents’ self-efficacy increases when they perceive support from significant others, including teachers (Brewster & Bowen, 2004; R. D. Liu et al., 2017). Moreover, various dimensions of teacher support—autonomy support, emotional support and academic support—enhance students’ academic self-efficacy. For instance, teachers’ autonomy support, by encouraging independent thinking and providing feedback, enhances students’ sense of competence (Luo et al., 2014), leading to higher academic self-efficacy. Their emotional support also builds students’ confidence (Sun et al., 2024), further boosting their belief in their learning abilities. This confidence is crucial, as students’ perception of competence influences task engagement. Additionally, an empirically based study on college students demonstrated that perceived academic support can enhance self-efficacy (Sheu et al., 2022).

As Job Demands–Resources (JD-R) Theory proposes, resources can be classified into dimensions like job-related and individual-related resources. The acquisition of job-related resources exerts a facilitative effect on the promotion of individual-related resources, and both of them collectively contribute to the enhancement of one’s job engagement (Demerouti et al., 2001). In an instructional setting, perceived teacher support functions as a vital social-environmental job resource. By providing encouragement and guidance, it can enhance students’ psychological personal resources (academic self-efficacy) through which it promotes positive academic engagement.

Research indicates that teacher support associates with advantageous teacher–student interaction, boosting students’ self-efficacy (J. Liu et al., 2025). Academic self-efficacy is an indispensable component of psychological capital. It is associated with decreased learning fatigue (Schaufeli et al., 2002b) and academic engagement favorably (Galyon et al., 2012).

Building upon findings, we advance the second hypothesis:

**H2:**  *Perceived teacher support contributes to students’ academic engagement*, *which is a process mediated by academic self-efficacy.*

### 1.3. Proactive Personality’s Moderating Role

Proactive personality refers to stable characteristics that motivate individuals to actively seek opportunities, overcome obstacles, and persist toward goal achievement despite challenges (Bateman & Crant, 1993). Research proves that proactive individuals identify opportunities, utilize resources, and actively alter their learning environments (S. K. Parker & Collins, 2010), thus resulting in increased academic engagement (Major et al., 2006). Therefore, proactive students typically have higher levels of academic engagement.

The COR’s gain spiral emphasizes that sufficient resources help individuals acquire additional ones, generating greater gains, which result in a positive feedback loop (Tian & Liu, 2024; Halbesleben & Wheeler, 2015). Thus, positive resources can intertwine with one another and yield cumulative positive outcomes. In academic contexts, an individual trait (proactive personality) and a cognitive resource (academic self-efficacy) can interact to shape students’ learning outcomes (academic engagement).

The Proactive Motivation Model suggests that differences in proactive motivation and the resulting action exhibit heterogeneity among individuals (S. K. Parker et al., 2010). Within this theoretical framework, highly proactive students tend to embrace active behaviors, which interact with their academic self-efficacy, thus amplifying the mediating effect.

Empirical studies also indicate that strong proactive traits amplify how cognitive factors positively influence academic outcomes. Proactive personality traits, in tandem with professional identity, foster pre-service teachers’ academic engagement (Yao & Xu, 2022). In the same vein, as a critical moderating variable, proactive personality can further reinforce academic self-efficacy’s positive role when driving academic engagement and ultimately reach elevated level’s academic engagement, enabling students to maximize the utilization of available resources.

Therefore, this study proposes that:

**H3:**  *Proactive personality among high school students exerts a moderating effect on how academic self-efficacy influences their academic engagement.*

**Figure 1:**
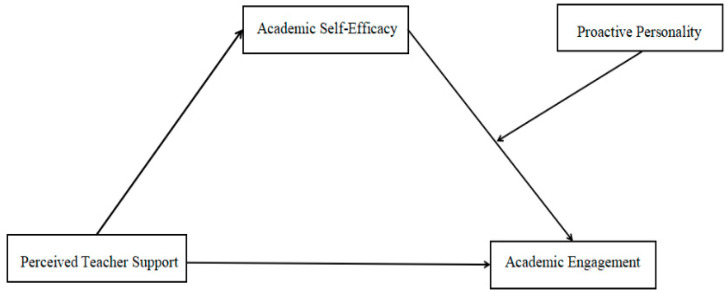
Hypothesis model.

To conclude, this study aims to propose a more comprehensive academic engagement paradigm. That is, different individual attributes shape how teacher support affects academic engagement, with proactive personality, a relatively stable trait, playing a key moderating role. Therefore, it unpacks the mechanism through which students’ perceived teacher support influences their academic engagement, where academic self-efficacy functions as a mediator and proactive personality assumes role of a moderator (Figure 1).

## 2. Materials and Methods

### 2.1. Participants

Cluster sampling approach was executed in the present research, with classes serving as a main selection unit. Approximately 1800 questionnaires were distributed to a public senior high school students ranging from grades 10 to 12 in Xinyang city located in southeastern Henan Province, China. After applying validity screening using lie-detection items, invalid responses (incomplete or incorrectly filled questionnaires) and abnormal responses (e.g., identical answers to all items) were removed. Ultimately, 1652 valid questionnaires were retained for analysis in this study, yielding an effective response rate of 91.8%. The responses included 718 males (43.1%) and 946 females (56.9%). Among them, 273 are freshmen (16.4%), 641 are second-year students (38.5%), and 750 are third-year students (45.1%).

### 2.2. Measures

#### 2.2.1. Perceived Teacher Support Scale

For assessing students’ perceptions of teacher support, this study adopted Student Perceived Teacher Support Behavior Scale, constructed by OuYang (2005). It includes learning, emotional, and ability support three factors, comprising 19 items like “The teacher encourages me with eye contact to stand up and answer questions.” We applied 6-point rating system. 1 signifies “completely disagree” while 6 means “completely agree.” The 11th item was scored in reverse. The higher the ratings, the more perceived support. Overall scale has strong internal consistency (Cronbach’α is 0.91). KMO = 0.93 and Bartlett’s test was significant (*p* < 0.001), indicating data suitability for factor analysis. Results from the confirmatory factor analysis (CFI ≈ 0.9, RMSEA ≈ 0.08, SRMR ≈ 0.05) indicated that the scale demonstrated satisfactory structural validity. The CR values were 0.82, 0.81, and 0.77, and the corresponding AVE values were 0.36, 0.42, and 0.46. Although some AVE values are below the ideal threshold of 0.50, the high CR and satisfactory CFA fit suggest that the three dimensions maintain acceptable reliability and convergent validity.

#### 2.2.2. Academic Engagement Scale

To evaluate high school students’ academic engagement, this study employed Fang et al.’s (2008) Academic Engagement Scale—a Chinese-adapted and revised version of Schaufeli et al.’s (2002a) original scale. It comprises vigor, dedication, and absorption dimensions, comprising 17 items, including “I can study for long periods without a break” and “When studying, I forget everything around me.” Responses were assessed using a 7-point Likert scale, with ratings ranging from 1 (never) to 7 (always); higher scores in this scale correspond to a higher level of academic engagement. Overall Cronbach’α score is 0.95. The data were deemed suitable for factor analysis, with a KMO value of 0.95 and a significant Bartlett’s test (*p* < 0.001). Results from the confirmatory factor analysis (CFI ≈ 0.96, RMSEA ≈ 0.06, SRMR ≈ 0.04) indicated that the scale demonstrated satisfactory structural validity. The CR values were 0.89, 0.89, and 0.90, and the corresponding AVE values were 0.56, 0.62, and 0.59, indicating good reliability and convergent validity for the three dimensions.

#### 2.2.3. Academic Self-Efficacy Scale

This study used Zeng’s (2008) Academic Self-Efficacy Scale, revised from Liang’s (2002) questionnaire. It comprises 18 items across two dimensions: learning ability and learning behavior. Responses were rated by 5-point Likert scale (1 = completely disagree, 5 = completely agree). Higher scores indicate stronger academic self-efficacy. The scale exhibited favorable reliability (Cronbach’s α = 0.90). Factor analysis was appropriate, as indicated by a KMO value of 0.93 and a significant Bartlett’s test (*p* < 0.001). The confirmatory factor analysis showed that the scale possessed adequate structural validity (CFI ≈ 0.94, RMSEA ≈ 0.06, SRMR ≈ 0.04). The CR values were 0.85 and 0.87, and the corresponding AVE values were 0.46 and 0.48. Although the AVE values are slightly below the ideal threshold of 0.50, the high CR and satisfactory CFA fit indicate that this scale still demonstrates adequate measurement quality.

#### 2.2.4. Proactive Personality Scale

Proactive personality was assessed using Shang and Gan’s (2009) revised Proactive Personality Scale, which includes 11 items. Researchers used 7-point Likert scale (1 representing strongly disagree and 7 representing strongly agree). Higher scores reflect stronger proactivity. The Cronbach’s α value is 0.842. The KMO value (0.88) and the significant result of Bartlett’s test (*p* < 0.001) indicated that the data were suitable for factor analysis. Confirmatory factor analysis further supported that the scale had satisfactory structural validity, with fit indices of CFI ≈ 0.92, RMSEA ≈ 0.08, and SRMR ≈ 0.04. The CR value was 0.85, and the corresponding AVE was 0.34. Despite the AVE being below 0.50, the high CR and acceptable CFA indices support the scale’s adequacy for subsequent analyses.

### 2.3. Data Collection and Analysis

Questionnaires were administered to class, with the administrators briefing participants on guidelines beforehand; the survey took 20 min, after which all completed forms were gathered.

Data analysis was conducted using SPSS 27.0 and Mplus 8.3. Prior to data analysis, normality tests were conducted for all primary variables of the scales. The results showed that the absolute values of skewness for all variables were less than 2, and the absolute values of kurtosis were less than 7, indicating that the distributions of the variables were approximately normal and met the assumptions for subsequent analyses. The Little’s MCAR test (*p* = 0.815) indicated that the data were consistent with the assumption of missing completely at random (MCAR). Therefore, missing data were handled using listwise deletion (Wen et al., 2018). Gender, class leadership status, and academic performance level were included as control variables and all variables were standardized. A mediation model with a moderator was tested, with the bootstrap approach (5000 resamples) employed to examine mediating effects.

### 2.4. Class/School Nesting and Robustness Check

Due to a cluster sampling design with students nested within classes/schools, potential clustering effects should be considered (Dong et al., 2016). However, class identifiers were not retained during data collection, making it impossible to use multilevel modeling for clustering in the main analyses. Therefore, following K. Parker et al. (2025), whose study showed that class-level intraclass correlation coefficients (ICCs) range from 0.011 to 0.035, we conducted a sensitivity analysis based on the design effect formula (DEFF = 1 + (m − 1) × ICC) to simulate the potential inflation of standard errors under class-level clustering (see Supplementary Materials). This analysis was used to evaluate the potential impact of clustering; the main analyses were reported using the original, unadjusted standard errors.

### 2.5. Ethical Considerations

Before conducting this survey, participants were furnished with written informed consent. The principle of voluntary participation was strictly followed, allowing them to withdraw from the study at any phase. Subsequently, researchers read out the instructions for questionnaire completion and clarified that the data gathered through this survey would be anonymized, maintained under strict confidentiality, and utilized only for research purposes. IRB approval was obtained on 1 September 2023.

## 3. Results

### 3.1. Common Method Bias (CMB) Test

We applied Harman’s single-factor for data validation and discovered that ten eigenvalues were greater than one, and the primary factor explained 28.9% of total variance, indicating no significant common method effects.

Moreover, several procedural remedies were implemented in the survey design to minimize potential CMB (Tang & Wen, 2020). Specifically, (1) lie-detection items and anonymous response protocols were integrated into the survey design. (2) Reverse-coded items were included within the same scale; (3) the order of scale items was randomized to mitigate potential sequence effects; (4) Attention-check items were randomly inserted to ensure careful responding (e.g., “Please select option 5 for this item”).

### 3.2. Descriptive Statistics and Correlation Analysis

To examine the bivariate relationships among key variables in high school student sample, we used Pearson Correlation Analysis. All variables showed significant intercorrelations (see Table 1).

### 3.3. The Mediating Role of Academic Self-Efficacy

**Figure 2:**
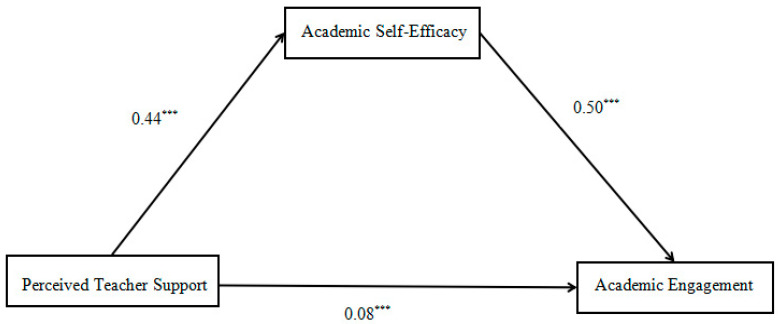
Mediation model (The model illustrates that academic self-efficacy mediates the relationship between perceived teacher support and learning engagement). *** *p* < 0.001.

We examined academic self-efficacy’s mediating role utilizing the PROCESS macro Model 4 (see Figure 2). The analytical results indicated that perceived teacher support was significantly associated with academic engagement and academic self-efficacy. When academic self-efficacy was included, perceived teacher support remained a significant predictor of academic engagement through academic self-efficacy (see Table 2).

Indirect effects were tested using a bias-corrected bootstrap procedure with 5000 resamples and a 95% confidence interval (CI). The findings confirmed, that academic self-efficacy served as a mediator. The 95% confidence interval [0.23, 0.29] further supports this mediation significance (Table 3).

### 3.4. The Moderating Role of Proactive Personality

To test proactive personality’s moderating effect on the latter pathway, the PROCESS Macro Model 14 was utilized, employing a bias-corrected bootstrap approach with 5000 resamples and a 95% CI. Before conducting the analysis, both academic self-efficacy and proactive personality were mean-centered to minimize potential multicollinearity. All variance inflation factor (VIF) values were well below the threshold of 5 (ranging from 1.0 to 1.5), suggesting no multicollinearity issue. The results demonstrated that academic self-efficacy and proactive personality jointly contributed to academic engagement (see Table 4).

**Figure 3:**
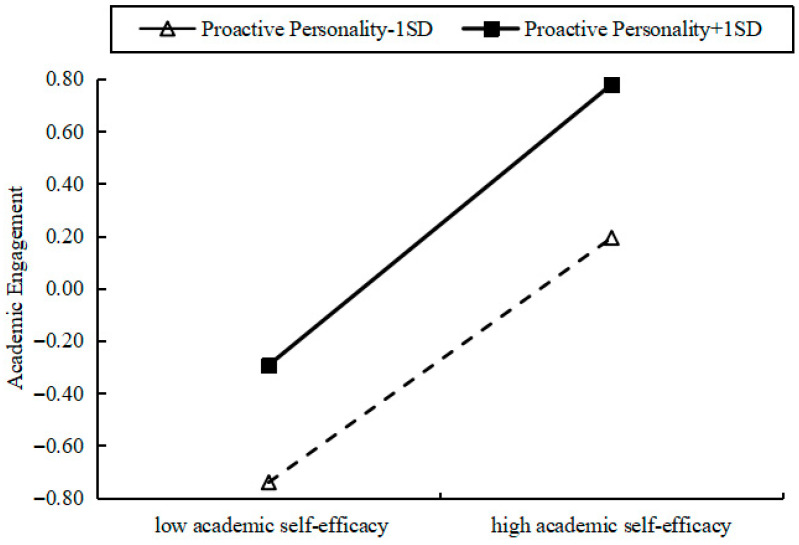
Simple slope.

Simple slope analyses were conducted by plotting academic self-efficacy against academic engagement, with proactive personality categorized into high and low groups to examine the moderating effect. The simple slopes were significant at all levels of proactive personality: low (−1*SD*, slope = 0.47, *t* = 17.39, *p* < 0.001), mean (slope = 0.50, *t* = 22.06, *p* < 0.001), and high (+1*SD*, slope = 0.54, *t* = 19.55, *p* < 0.001) (see Table 5 and Figure 3). Johnson-Neyman (J-N) analysis showed that across the full observed range of standardized proactive personality scores (−3.86 to 1.8782), no transition points were detected, suggesting that the effect of academic self-efficacy on learning engagement remained significant at all levels of proactive personality (all *p* < 0.001, 95% CIs do not include 0). Overall, the results showed that the positive association between academic self-efficacy and academic engagement strengthened as proactive personality increased.

## 4. Discussion

This research investigated the ways in which perceived teacher support influences high school students’ academic engagement. Findings lend confirmation to a pronounced positive connection between perceived teacher support and academic engagement, with academic self-efficacy serving as an intermediary variable. Notably, proactive personality was found to moderate this mediating process.

### 4.1. Perceived Teacher Support Positively Is Associated with Students’ Academic Engagement

The current research verified Hypothesis 1 that perceived teacher support predicted students’ academic engagement positively, which is consistent with prior studies (Shao et al., 2025; Sadoughi & Hejazi, 2021; Guo et al., 2023). Congruent with SDT, the findings state that adequate social support fulfills individuals’ needs for competence, belonging, and other basic psychological needs, hence encouraging positive behaviors. Among various forms of social support, teachers play a particularly crucial role for students. Teachers’ support aids in fulfilling these needs, where the emotional and cognitive dimensions of perceived teacher support contribute to academic engagement. For example, appreciation from teachers provides positive feedback to learners, raising their learning interest and motivation to engage in academic assignments (Federici & Skaalvik, 2014; Li et al., 2019). Additionally, teachers’ cognitive support, such as instructional scaffolding in mathematics, helps students solve problems and foster their engagement (Chen et al., 2024). Teachers’ recognition of students’ abilities encourages them to accept challenges, reduces anxiety, and helps them engage more deeply in learning. Overall perceived teacher support, in turn, delivers emotional sustenance and cognitive scaffolding, and teacher expectations motivate students to elevate competence, resulting in enhanced academic engagement.

### 4.2. Mediating Function of Academic Self-Efficacy

The present research delineated the function of academic self-efficacy, which operates as a partial mediator in how perceived teacher support is associated with academic engagement. Aligning with Social Cognitive Theory, which suggests environmental and individual cognitive factors jointly shape behavior, the results posit that perceived teacher support finds its pathway to students’ academic engagement via their academic self-efficacy. More specifically, teachers’ positive feedback, serving as an influential factor (i.e., verbal persuasion) of self-efficacy, improves students’ perceived competence in task execution (Bandura, 1986). Learners with robust self-efficacy invest greater effort to succeed, boosting academic accomplishment and engagement (El-Sayad et al., 2021).

The finding also converges with empirical studies documenting a linkage between external support and student achievement (Huang & Liu, 2025). For instance, graduate students’ academic engagement is indirectly affected by social support. The pathway for indirect impact lies in academic self-efficacy (Yu et al., 2024).

### 4.3. Moderating Role of Proactive Personality

The current research concluded that proactive personality strengthens the mediating function of academic self-efficacy, thereby validating Hypothesis 3. Higher proactive students demonstrate a more heightened propensity to advance their academic engagement through academic self-efficacy. This is consistent with “resource gain spiral” in COR Theory, where perceived teacher support, as a form of external resource, can be transformed into internal resources (self-efficacy). The enrichment of internal resources further motivates individuals to convert resource advantages into more sustained engagement, forming a positive spiral upward cycle (Halbesleben & Wheeler, 2015; Yuan et al., 2026). Proactive personality acts as an “accelerator” in this process. Proactive students are more adept at actively seeking and utilizing resources such as teacher support, which enables them to convert these resources into self-efficacy and partake in challenging learning tasks to accelerate the operation of resource gain spiral.

This result also confirms Fergus and Zimmerman’s (2005) enhancement hypothesis, which posits that a protective factor (i.e., proactive personality in this study) amplifies how one variable (academic self-efficacy) influences outcome variables (academic engagement). This hypothesis is further corroborated by Su et al. (2024), who found that harmonious teacher–student interactions are positively associated with graduate students’ creative academic self-efficacy, and that proactive personality strengthens this predictive relationship.

## 5. Conclusions

This study reached the following conclusions. First, perceived teacher support plays a substantial positive role in students’ engagement in academic activities, indicating that environmental factors contribute to shaping students’ learning behaviors. In addition, academic self-efficacy operates as a mediating factor within this relational dynamic: when students perceive teacher support, they develop higher academic self-efficacy, forming a firm belief in their learning competence. This confidence further motivates them to participate in the learning process and makes them more engaged. Finally, this study underscores the constructive enhancing impact of proactive personality (as a moderator) on fostering academic engagement. It also sheds light on our understanding of the boundary conditions under which perceived teacher support promotes academic engagement.

Theoretically, this research lends support to and extends the realm of JD-R Theory’s and COR Theory’s application in educational settings. Perceived teacher support (a critical external resource) facilitates academic engagement by enhancing academic self-efficacy (a core internal resource). This finding validates both JD-R Theory’s “motivational process” and COR Theory’s “resource gain spiral”. Individuals with proactive personalities demonstrate greater enthusiasm in seeking out and investing in such resources, which speeds up the resource transformation process and fosters positive resource accumulation. These results suggest that educational interventions ought to pay equal attention to constructing a supportive environment and to nurturing proactive personalities, maximizing academic engagement through the collaborative effect of internal and external resources.

Practically, the present study provides insights for high school teaching. To start with, teachers are advised to guide students in boosting their academic self-efficacy. For example, teachers can use diverse evaluations to affirm students’ efforts. Second, to cultivate students’ proactive personalities within educational settings, teaching methods should be tailored to students’ individual differences. For students with highly proactive personalities, teachers should provide greater opportunities for independent learning and expression. In contrast, for students with less proactive personalities, teachers should adopt strategies to nurture their proactivity, as it plays a vital role in enhancing academic engagement. For example, teachers can use group learning methods to encourage peer cooperation and stimulate proactive behaviors in the classroom. Moreover, teachers are encouraged to update their educational philosophy, and avoid using grades as the sole criterion for evaluating students’ progress. However, as the data were collected from a single public high school system in one region, the generalizability of the findings may be more applicable to educational contexts with similar school settings.

Despite the contributions of the present study, several limitations should be acknowledged, along with suggestions for future research. For one, cross-sectional analysis cannot determine causal links between variables. Subsequent research might employ a longitudinal design, following the same participant cohort over a prolonged time frame to capture temporal changes. Second, the study focused solely on high school students from specific regions of China. To increase the external validity of these findings, future study should incorporate samples across diverse regions, educational stages, and cultural contexts. Third, there was a lack of certain environmental (such as family support), institutional (such as evaluation system), and individual-level factors (such as optimistic traits) that may affect learning engagement. Future studies could consider examining a broader range of influences across different ecological settings, along with other personal characteristics to achieve a more thorough understanding of student engagement. In addition, other variables, such as grade, gender and socioeconomic status, can be incorporated into multi-group analyses to investigate whether these factors shape the relationships among the key variables. Last, our study used the class-level cluster sampling without implementing multilevel modeling or cluster-robust standard error corrections. This means that potential effects arising from the nesting of students within classes or schools were not statistically accounted for, which may have affected the accuracy of standard errors and significance tests. Future research should collect class-level or school-level information to enable more precise multilevel analyses.

## Figures and Tables

**Table 1:** Correlation Analysis.

Variables	*M*	*SD*	1	2	3	4
PTS	3.54	0.86	1			
ASE	2.90	0.71	0.51 ***	1		
Proactive Personality	5.36	0.87	0.41 ***	0.45 ***	1	
Academic Engagement	4.16	1.16	0.43 ***	0.63 ***	0.51 ***	1

*** *p* < 0.001.

**Table 2:** Regression Analysis.

Regression Equation		Model Fit				Regression Coefficients and Significance			
Outcome Variable	Predictor Variable	*R*	*R* ^2^	*F*	Δ*R* ^2^	*β*	*SE*	*t*	95% CI
Academic Engagement	PTS	0.45	0.21	107.29 ***		0.40	0.02	17.48 ***	[0.35, 0.44]
ASE	gender					−0.2	0.04	−5.03 ***	[−0.29, −0.13]
	academic performance level					−0.22	0.01	−12.29 ***	[−0.26, −0.19]
	class leadership status					−0.003	0.008	−0.42	[−0.02, 0.01]
	PTS	0.58	0.34	209.32 ***		0.44	0.02	21.17 ***	[0.40, 0.48]
Academic Engagement	gender					0.26	0.04	6.89 ***	[0.19, 0.34]
	academic performance level					0.002	0.02	0.12	[−0.03, 0.04]
	class leadership status					0.004	0.008	0.60	[−0.01, 0.02]
	PTS	0.66	0.43	251.29 ***	0.21	0.14	0.02	6.45 ***	[0.09, 0.18]
	ASE					0.58	0.02	25.62 ***	[0.54, 0.63]

Note: Δ*R*^2^ represents the change in explained variance from the previous model. *** *p* < 0.001.

**Table 3:** Direct and Indirect Effect Tests.

	Mediation Effect Path	Effect Value	Boot *SE*	Boot 95% CI	Effect Size (%)
Total Effect	PTS → Academic Engagement	0.40	0.02	[0.35, 0.44]	
Direct Effect	PTS → Academic Engagement	0.14	0.02	[0.09, 0.18]	35%
Indirect Effect	PTS → ASE → Academic Engagement	0.26	0.02	[0.23, 0.29]	65%

**Table 4:** Moderating effect test of proactive personality.

Regression Equation		Model Fit			Regression Coefficients and Significance			95% CI	
Outcome Variable	Predictor Variable	*R*	*R* ^2^	*F*	*β*	*SE*	*t*	LLCI	ULCI
ASE	gender				−0.21	0.04	−5.03 ***	−0.29	−0.13
	academic performance level				−0.22	0.02	−12.27 ***	−0.26	−0.19
	class leadership status				−0.004	0.0008	−0.42	−0.02	0.01
	PTS	0.58	0.34	209.32 ***	0.44	0.02	21.17 ***	0.40	0.48
	gender				0.22	0.04	6.16 ***	0.15	0.29
	academic performance level				0.003	0.02	0.20	−0.03	0.04
	class leadership status				0.003	0.001	0.44	−0.01	0.02
Academic Engagement	PTS	0.70	0.48	220.93 ***	0.08	0.02	3.66 ***	0.04	0.12
	ASE				0.50	0.02	21.84 ***	0.45	0.54
	Proactive Personality				0.26	0.02	12.71 ***	0.22	0.30
	ASE × Proactive Personality				0.04	0.01	2.39 **	0.006	0.065

*** *p* < 0.001, ** *p* < 0.01.

**Table 5:** The Moderating Effect of Proactive Personality.

	Proactive Personality	Effect Value	Boot *SE*	95% CI
Mediating Effect of ASE	*M* − 1*SD*	0.47	0.03	[0.41, 0.52]
	*M*	0.50	0.02	[0.46, 0.55]
	*M* + 1*SD*	0.54	0.02	[0.48, 0.59]

## Data Availability

The raw data supporting the conclusions of this article will be made available by the authors upon request.

## Supplementary Materials

The following supporting information can be downloaded at: https://www.mdpi.com/article/10.3390/bs15111594/s1.

## References

[B1-behavsci-15-01594] Ahmed W., Minnaert A., van der Werf G., Kuyper H. (2010). Perceived social support and early adolescents’ achievement: The mediational roles of motivational beliefs and emotions. Journal of Youth and Adolescence.

[B2-behavsci-15-01594] Babad E. (1990). Measuring and changing teachers’ differential behavior as perceived by students and teachers. Journal of Educational Psychology.

[B3-behavsci-15-01594] Bakker A. B., Demerouti E., Euwema M. C. (2005). Job resources buffer the impact of job demands on burnout. Journal of Occupational Health Psychology.

[B4-behavsci-15-01594] Bandura A. (1986). The explanatory and predictive scope of self-efficacy theory. Journal of Social and Clinical Psychology.

[B5-behavsci-15-01594] Bateman T. S., Crant J. M. (1993). The proactive component of organizational behavior: A measure and correlates. Journal of Organizational Behavior.

[B6-behavsci-15-01594] Brewster A. B., Bowen G. L. (2004). Teacher support and the school engagement of Latino middle and high school students at risk of school failure. Child and Adolescent Social Work Journal.

[B7-behavsci-15-01594] Bronfenbrenner U., Evans G. W. (2000). Developmental science in the 21st century: Emerging questions, theoretical models, research designs and empirical findings. Social Development.

[B8-behavsci-15-01594] Chen T., Chen L., Chu Y., Sun Q. (2024). The effect of teacher support on students’ mathematics learning engagement: Take Xizang students in three areas as an example. Teacher Education Research.

[B9-behavsci-15-01594] Cong Y., Yang L., Proietti Ergün A. L. (2024). Exploring the relationship between burnout, learning engagement and academic self-efficacy among EFL learners: A structural equation modeling analysis. Acta Psychologica.

[B10-behavsci-15-01594] Deci E. L., Ryan R. M., Liu W., Wang J., Ryan R. (2016). Optimizing students’ motivation in the era of testing and pressure: A self-determination theory perspective. Building autonomous learners.

[B11-behavsci-15-01594] Demerouti E., Bakker A. B., Nachreiner F., Schaufeli W. B. (2001). The job demands-resources model of burnout. Journal of Applied Psychology.

[B12-behavsci-15-01594] Dong N., Reinke W. M., Herman K. C., Bradshaw C. P., Murray D. W. (2016). Meaningful effect sizes, intraclass correlations, and proportions of variance explained by covariates for planning two- and three-level cluster randomized trials of social and behavioral outcomes. Evaluation Review.

[B13-behavsci-15-01594] El-Sayad G., Saad N. H. M., Thurasamy R. (2021). How higher education students in Egypt perceived online learning engagement and satisfaction during the COVID-19 pandemic. Journal of Computers in Education.

[B14-behavsci-15-01594] Fall A. M., Roberts G. (2012). High school dropouts: Interactions between social context, self perceptions, school engagement, and student dropout. Journal of Adolescence.

[B15-behavsci-15-01594] Fang L. T., Shi K., Zhang F. H. (2008). Research on reliability and validity of utrecht work engagement scale-student. Chinese Journal of Clinical Psychology.

[B16-behavsci-15-01594] Federici R. A., Skaalvik E. M. (2014). Students’ perceptions of emotional and instrumental teacher support: Relations with motivational and emotional responses. International Education Studies.

[B17-behavsci-15-01594] Fergus S., Zimmerman M. A. (2005). Adolescent resilience: A framework for understanding healthy development in the face of risk. Annual Review of Public Health.

[B18-behavsci-15-01594] Galyon C. E., Blondin C. A., Yaw J. S., Nalls M. L., Williams R. L. (2012). The relationship of academic self-efficacy to class participation and exam performance. Social Psychology of Education.

[B19-behavsci-15-01594] García-Moya I., Díez M., Paniagua C. (2023). Stress of school performance among secondary students: The role of classroom goal structures and teacher support. Journal of School Psychology.

[B20-behavsci-15-01594] Guo Q., Samsudin S., Yang X., Gao J., Ramlan M. A., Abdullah B., Farizan N. H. (2023). Relationship between perceived teacher support and student engagement in physical education: A systematic review. Sustainability.

[B21-behavsci-15-01594] Halbesleben J. R. B., Wheeler A. R. (2015). To invest or not? The role of coworker support and trust in daily reciprocal gain spirals of helping behavior. Journal of Management.

[B22-behavsci-15-01594] Huang T., Liu S. (2025). The relationship between perceived teacher emotional support and behavioral engagement: The chain mediating effect of self-efficacy and academic resilience. Modern Foreign Languages.

[B23-behavsci-15-01594] Jin G., Wang Y. (2019). The influence of gratitude on learning engagement among adolescents: The multiple mediating effects of teachers’ emotional support and students’ basic psychological needs. Journal of Adolescence.

[B24-behavsci-15-01594] Lewis A. D., Huebner E. S., Malone P. S., Valois R. F. (2011). Life satisfaction and student engagement in adolescents. Journal of Youth and Adolescence.

[B25-behavsci-15-01594] Li X., Qiao H., Liu Y., Gao D. (2019). Perceived teachers’ emotional support and learning burnout in middle school students: A mediated moderation model. Chinese Journal of Clinical Psychology.

[B26-behavsci-15-01594] Liang Y. S. (2002). Study on achievement goals, attribution styles and academic self-efficacy of college students. Unpublished master’s thesis.

[B28-behavsci-15-01594] Liu J., Gao J., Arshad M. H. (2025). Teacher-student relationships as a pathway to sustainable learning: Psychological insights on motivation and self-efficacy. Acta Psychologica.

[B27-behavsci-15-01594] Liu R. D., Zhen R., Ding Y., Liu Y., Wang J., Jiang R., Xu L. (2017). Teacher support and math engagement: Roles of academic self-efficacy and positive emotions. Educational Psychology.

[B29-behavsci-15-01594] Liu Z. H. (2016). On the effect of secondary school students’ academic stress on their learning engagement: The mediating effect of academic resilience. Chinese Journal of Special Education.

[B30-behavsci-15-01594] Luo Y., Zhao M., Wang Z. (2014). Effect of perceived teacher’s autonomy support on junior middle school students’ academic burnout: The mediating role of basic psychological needs and autonomous motivation. Psychological Development and Education.

[B31-behavsci-15-01594] Major D. A., Turner J. E., Fletcher T. D. (2006). Linking proactive personality and the Big Five to motivation to learn and development activity. Journal of Applied Psychology.

[B32-behavsci-15-01594] Olivier E., Archambault I., De Clercq M., Galand B. (2019). Student self-efficacy, classroom engagement, and academic achievement: Comparing three theoretical frameworks. Journal of Youth and Adolescence.

[B33-behavsci-15-01594] Ouyang D. (2005). A research on the relation among teachers’ expectation, self-conception of students’ academic achievement, students’ perception of teacher’s behavioral supporting and the study achievement. Master’s thesis.

[B34-behavsci-15-01594] Parker K., Nunns M., Xiao Z. M., Ford T., Stallard P., Kuyken W., Axford N., Ukoumunne O. C. (2025). Patterns of intra-cluster correlation coefficients in school-based cluster randomised controlled trials of interventions for improving social-emotional functioning outcomes in pupils: A secondary data analysis of five UK-based studies. BMC Medical Research Methodology.

[B36-behavsci-15-01594] Parker S. K., Bindl U. K., Strauss K. (2010). Making things happen: A model of proactive motivation. Journal of Management.

[B35-behavsci-15-01594] Parker S. K., Collins C. G. (2010). Taking stock: Integrating and differentiating multiple proactive behaviors. Journal of Management.

[B37-behavsci-15-01594] Reddy R., Rhodes J. E., Mulhall P. (2003). The influence of teacher support on student adjustment in the middle school years: A latent growth curve study. Development and Psychopathology.

[B38-behavsci-15-01594] Robayo-Tamayo M., Blanco-Donoso L. M., Román F. J., Carmona-Cobo I., Moreno-Jiménez B., Garrosa E. (2020). Academic engagement: A diary study on the mediating role of academic support. Learning and Individual Differences.

[B39-behavsci-15-01594] Rooij E. V., Jansen E. P., Grift W. V. (2017). Secondary school students’ engagement profiles and their relationship with academic adjustment and achievement in university. Learning and Individual Differences.

[B40-behavsci-15-01594] Sadoughi M., Hejazi S. Y. (2021). Teacher support and academic engagement among EFL learners: The role of positive academic emotions. Studies in Educational Evaluation.

[B41-behavsci-15-01594] Schaufeli W. B., Martínez I. M., Marques Pinto A., Salanova M., Bakker A. B. (2002). Burnout and engagement in university students: A cross-national study. Journal of Cross-Cultural Psychology.

[B42-behavsci-15-01594] Schaufeli W. B., Salanova M., González-Romá V., Bakker A. B. (2002). The measurement of engagement and burnout: A two sample confirmatory factor analytic approach. Journal of Happiness Studies: An Interdisciplinary Forum on Subjective Well-Being.

[B43-behavsci-15-01594] Shang J. Y., Gan Y. Q. (2009). Analysis of the effects of the proactive personality on graduates’ career decision-making self-efficacy. Acta Scientiarum Naturalium Universitatis Pekinensis.

[B44-behavsci-15-01594] Shao Y., Feng Y., Zhao X., Liu G., Zhang L. (2025). Teacher support and secondary school students’ learning engagement: A moderated mediation model. Scientific Reports.

[B45-behavsci-15-01594] Sheu H. B., Chong S. S., Dawes M. E. (2022). The chicken or the egg? Testing temporal relations between academic support, self-efficacy, outcome expectations, and goal progress among college students. Journal of Counseling Psychology.

[B46-behavsci-15-01594] Shumaker S. A., Brownell A. (1984). Toward a theory of social support: Closing conceptual gaps. Journal of Social Issues.

[B47-behavsci-15-01594] Strati A. D., Schmidt J. A., Maier K. S. (2017). Perceived challenge, teacher support, and teacher obstruction as predictors of student engagement. Journal of Educational Psychology.

[B48-behavsci-15-01594] Su W., Zhang Y., Yin Y., Dong X. (2024). The influence of teacher-student relationship on innovative behavior of graduate student: The role of proactive personality and creative self-efficacy. Thinking Skills and Creativity.

[B49-behavsci-15-01594] Sun W., Liu X., Liu A. (2024). Impact of perceived teacher emotional support on junior middle school students’ academic well-being: A mediated model with moderation. Journal of Beijing Institute of Education.

[B50-behavsci-15-01594] Tang D., Wen Z. (2020). Statistical approaches for testing common method bias: Problems and suggestions. Journal of Psychological Science.

[B51-behavsci-15-01594] Tian G. X., Liu T. (2024). Research on the resilience status and improvement measures of trainee teachers from the perspective of conservation of resources theory. Teacher Development Research.

[B52-behavsci-15-01594] Wen Z. L., Huang B. B., Tang D. D. (2018). Preliminary work for questionnaire data modeling. Journal of Psychological Science.

[B53-behavsci-15-01594] Wigfield A., Eccles J. S. (2000). Expectancy–value theory of achievement motivation. Contemporary Educational Psychology.

[B54-behavsci-15-01594] Wong Z. Y., Liem G. A. D., Chan M., Datu J. A. D. (2024). Student engagement and its association with academic achievement and subjective well-being: A systematic review and meta-analysis. Journal of Educational Psychology.

[B55-behavsci-15-01594] Yao Q., Xu Y. (2022). Professional identification and learning engagement of targeted normal school students: The mesomeric effect of psychological capital and the regulating effect of initiative personality. Journal of Anhui University of Technology (Social Sciences).

[B56-behavsci-15-01594] Yu S. T., Song X. Y., Hu A. X., Zhao L. J., Shi X. Y. (2024). Relationship between perceived social support and special education postgraduates’ learning engagement: The chain mediating effect of professional identity and academic self-efficacy. Psychological Research.

[B57-behavsci-15-01594] Yuan Y., Li G., Liu J., Zhu Z., Zhang S. (2026). Leadership performance expectation on employee work outcomes: From the perspective of conservation of resources theory. Journal of Industrial Engineering and Engineering Management.

[B58-behavsci-15-01594] Zeng X. H. (2008). Senior school students’ academic self-efficacy and the relationship among academic self-efficacy, coping styles and mental health of senior school students. Unpublished master’s thesis.

[B59-behavsci-15-01594] Zhou A., Hu Y., Liu J. T., Lu X., Wang Y., Zhou Y. (2022). Relationship between social anxiety and academic engagement: The mediating role of intentional self-regulation and the age difference. Psychological Development and Education.

