# Supplementary material for: Does Teacher Support Scaffold Engagement? Academic Self-Efficacy as Mediator and Proactive Personality as Moderator Among Chinese High School Students
Source: Behav Sci (Basel). 2025 Nov 20;15(11):1594. doi: 10.3390/bs15111594 (PMC12649727; doi:10.3390/bs15111594)
Supplement: Supplementary file 1 [file behavsci-15-01594-s001.zip › behavsci-3922938-supplementary.pdf]

Table 1. Moderating effect test of proactive personality without control variables

Regression Equation		Model Fit			Regression Coefficients and Significance			95%CI	
Outcome Variable	Predictor Variable	R	R^2	F	β	SE	t	LLCI	ULCI
ASE	PTS	0.51	0.26	585.06***	0.51	0.02	24.19***	0.47	0.55
Academic Engagement	PTS	0.69	0.47	369.41***	0.07	0.02	3.46***	0.04	0.12
	ASE				0.48	0.02	21.74***	0.43	0.52
	Proactive Personality				0.27	0.02	12.71***	0.23	0.31
	ASE×Proactive Personality				0.03	0.02	2.27**	0.0047	0.063
